# Trajectories of eating behavior during COVID-19 lockdown: Longitudinal analyses of 22,374 adults

**DOI:** 10.1016/j.clnesp.2021.01.046

**Published:** 2021-04

**Authors:** Moritz Herle, Andrea D. Smith, Feifei Bu, Andrew Steptoe, Daisy Fancourt

**Affiliations:** a Department of Biostatistics & Health Informatics, King's College London, London, UK; b Department of Behavioural Science and Health, University College London, London, UK

**Keywords:** Covid-19, Mental health, Eating behavior, Lockdown, Covid-19 social study

## Abstract

**Background & aims:**

The COVID-19 pandemic has led to the implementation of stay-at-home and lockdown measures. It is currently unknown if the experience of lockdown leads to long term changes in individual's eating behaviors. The objectives of this study were: i) to derive longitudinal trajectories of change in eating during UK lockdown, and ii) to identify risk factors associated with eating behavior trajectories.

**Method:**

Data from 22,374 UK adults from the UCL COVID-19 Social study (a panel study collecting weekly data during the pandemic) were analyzed from 28th March to 29th May 2020. Latent Class Growth Analysis was used to derive trajectories of change in eating. These were then associated with prior socio-economic, health-related and psychological factors using multinomial regression models.

**Results:**

Analyses suggested five trajectories, with the majority (64%) showing no change in eating. In contrast, one trajectory was marked by persistently eating more, whereas another by persistently eating less. Overall, participants with greater depressive symptoms were more likely to report any change in eating. Loneliness was linked to persistently eating more (OR = 1.07), whereas being single or divorced, as well as stressful life events, were associated with consistently eating less (OR = 1.69). Overall, higher education status was linked to lower odds of changing eating behavior (OR = 0.54–0.77). Secondary exploratory analyses suggest that participants self-reported to have overweight were more commonly categorised into the group consistently eating more, whereas participants with underweigh persistently ate less.

**Conclusion:**

In this study, we found that one third of the sample report changes in quantities eaten throughout the first UK lockdown period. Findings highlight the importance of adjusting public health programs to support eating behaviors in future lockdowns both in this and potential future pandemics. This is particularly important as part of on-going preventive efforts to prevent nutrition-related chronic diseases.

## 1 Introduction

On the 11th of March 2020, the World Health Organization declared the Coronavirus disease (COVID-19) outbreak a pandemic. On the 23rd of March, the UK government announced a nationwide lockdown, restricting freedom of movement outside of the home except for limited purposes such as purchasing essential items. These measures hugely impacted all aspects of life and resulted in disruption of normal routines and systems of support.

The psychological and economic impact of lockdown raised concerns early in the pandemic [1]. Quarantine has previously been associated with substantial and wide-ranging adverse mental health effects, including depression and anxiety [2]. However, how such disruption might affect overall eating behaviors, beyond the immediate concerns of food access, is less understood. Some previous cross-sectional studies have suggested that home confinement during the COVID-19 pandemic resulted in increased numbers of meals [3] and snacking [4]; whereas others have found that social-distancing and staying at home was associated with adherence to healthier diets, potentially due to the increase of home cooked meals [5]. However, understanding the potential lasting effects of lockdown measures on eating behaviors, and consequentially on body weight, are crucial. This is not only for general well-being and population health but crucially also because having overweight and obesity are risk factors for hospitalization and severe illness progression of COVID-19 [6].

Hence, the continued focus on non-communicable disease (NCD) prevention and management are key public health policy priorities during the COVID-19 crisis and in potential future epidemics. If the response during outbreaks is not adapted to incorporate prevention and management of NCD risks, many people will suffer at a time when their vulnerability is already heightened [7]. Due to the close links between nutrition and health, it is essential to identify the risk factors that predispose individuals at risk of experiencing detrimental effects on their eating behaviors during government-issued lockdowns.

So far, previous research has used cross-sectional data, only providing a snapshot of eating behaviors at one time during lockdown due to the COVID-19 pandemic. Therefore, in this study we aimed to: i) describe how people's eating behavior (eating more, eating less, eating the same) changed over 8 weeks of lockdown in the UK, and ii) examine factors associated with distinct eating behavior trajectories.

## 2 Methods

### 2.1 Participants

Data were drawn from the COVID-19 Social Study; a large panel study of the psychological and social experiences of over 70,000 adults (aged 18+) in the UK during the COVID-19 pandemic. The study commenced on 21st March 2020 and involves online weekly data collection from participants for the duration of the COVID-19 pandemic in the UK. The study did not use a random sample and therefore is not representative of the UK population. However, it does contain a heterogeneous sample that was recruited using three primary approaches. First, snowballing was used, including promoting the study through existing networks and mailing lists (including large databases of adults who had previously consented to be involved in health research across the UK), print and digital media coverage, and social media. Second, more targeted recruitment was undertaken focusing on (i) individuals from a low-income background, (ii) individuals with no or few educational qualifications, and (iii) individuals who were unemployed. Third, the study was promoted via partnerships with third sector organisations to vulnerable groups, including adults with pre-existing mental health conditions, older adults, carers, and people experiencing domestic violence or abuse. The study was approved by the UCL Research Ethics Committee [12,467/005] and all participants gave informed consent.

For these analyses, we covered the time period beginning Friday 28th March until Thursday 4th of June 2020. This period spans the commencement of lockdown measures, introduced on 23rd March, and the lifting of strictest measures on 1st June. For our baseline predictor variables, we chose data collected during the week starting on the 28th of March, a week into lockdown. For the eating behavior measures, we chose the week commencing on Saturday 4th April 2020, as the studied question on eating behavior related to the prior week, and we wanted to ensure that this week in question fell into the lockdown period. To select our analyses sample we included participants who had data on the included predictors measured between 28th March-3rd April (N = 24,988) and had at least one measure of eating behavior between 4th April and 29th May (N = 62,046). The resulting analyses sample consisted of 22,374 participants.

### 2.2 Measures

#### 2.2.1 Primary outcome: eating behavior during lockdown

Participants were asked each week to indicate if they experienced a change in their eating over the past week: “Over the past week have you eating more than usual?”. The response options ranged from “less than usual”, “about the same” to “more than usual”.

#### 2.2.2 Sociodemographic characteristics

Standard sociodemographic data were collected in the baseline questionnaire. Age was derived from self-reported year of birth and categorized into age groups, 18–29, 30–45, 46–59 and 60+. Gender was reported as either male, female or other/prefer not to say. Marital status was grouped into “living with partner”, “living without partner”, “single”, or “divorced or widowed”. Ethnicity was dichotomized into “white” and “ethnic minority”. Marital status and living situation were grouped into four categories: “single, never married”, “single, divorced or widowed”, “in a relationship/married but living apart”, or “in a relationship/married and cohabiting”. Educational attainment was measured as the highest achieved level of education, ranging from “General Certificate of Secondary Education (GCSE) or below”, “A-levels or equivalent” to “Degree or above”. Usual total annual household income was dichotomized into “Low income < £30,000” or “≥ £30,000”.

#### 2.2.3 Mental health-related and psychological factors

Depressive symptoms during the past week were measured using the Patient Health Questionnaire (PHQ-9); a standard instrument for diagnosing depression in primary care [1]. The questionnaire includes nine items, with responses ranging from “not at all” to “nearly every day”. A higher score indicate more depressive symptoms.

Anxiety during the past week was measured using the Generalised Anxiety Disorder assessment (GAD-7); a well-validated tool used to screen and diagnose generalised anxiety disorder in clinical practice and research. The GAD-7 includes 7 items with 4-point responses ranging from “not at all” to “nearly every day”, with higher overall scores indicating more symptoms of anxiety [8].

Loneliness was measured using the 3-item UCLA-3 Loneliness Scale, a short form of the Revised UCLA Loneliness Scale (UCLA-R). Each item is rated with a 4-point rating scale, ranging from “never” to “always”, with higher scores indicating greater loneliness [9].

Social support was measured using an adapted version of the six-item short form of Perceived Social Support Questionnaire (F-SozU K-6) [10,11]. Each item is rated on a 5-point scale, with reponse options ranging from “not true at all” to “very true”. Higher scores indicate higher levels of perceived social support. Minor adaptations were made to the language in the scale to make it relevant to experiences during COVID-19 (see Supplementary Table 1 for a comparison of changes).

Participants were also asked to indicate to what extent they were worrying about COVID-19 specific topicsrtanging . Worries included the fear of not being able to access food, catching COVID-19, and becoming seriously ill. Minor and major worries were combined into one variable.

#### 2.2.4 Diagnosed physical and mental illness

Participants were asked if they had been diagnosed with a longstanding physical health condition (high blood pressure, diabetes, heart disease, lung disease or any other chronic physical health condition) or a clinically-diagnosed mental health problem (depression, anxiety or any other clinically-diagnosed psychiatric condition). Both were treated as binary variables.

#### 2.2.5 Body weight status

In the week starting 23rd May 2020, participants were asked to report their weight status prior to the introduction of lockdown measures: “How would you describe your weight usually (i.e. before lockdown started)?” The response options were "underweight", "normal weight", "slightly overweight", and "very overweight".

The full study protocol and an overview of collected information as part of this study (e.g. recruitment, retention, data cleaning, weighting and sample demographics) can be accessed at www.covidsocialstudy.org.

### 2.3 Analysis

#### 2.3.1 Latent trajectory modelling

Latent class growth analysis (LCGA) was used to derive trajectories of change in eating across ten weeks of lockdown (dates 4th April – 29th May). This method, an adapted form of the standard growth mixture model, is often used for categorical or ordinal data, as manifest variables are not assumed to be normally distributed [12,13]. We included linear and quadratic slopes in our model.

LCGA is an iterative process, whereby the number of underlying trajectories (or classes) is unknown and hence increased in each iteration. These alternative specifications are then compared using model fit indicators: Akaike information criterion (AIC), Bayesian information criterion (BIC), and adjusted BIC, with lowest values indicating better fit. Further, we report the entropy, which indicates the closeness of fit between the observed data and estimated trajectories, with high values being preferable. In addition, the number of participants per trajectory is considered, aiming to avoid trajectories with less than 3% of the sample. After selection of the best fitting model, estimations were repeated using 1000 random starts to avoid local maxima.

#### 2.3.2 Association with the predictors

Once the best fitting number of trajectories was selected, we examined the associations between socio-economic, health-related and psychological factors in the week starting 28th March and trajectory membership using the 3-step approach [14]. This method incorporates the posterior predicted probabilities for all classes from step 1 into the analyses linking predictors to trajectories. This way the uncertainty around class assignment is taken into consideration, increasing the reliability of the results [14]. Analyses were conducted using full information maximum likelihood, assuming missing at random. Results are reported as odds ratios in reference to a normative reference category. Alpha level was adjusted to account for the number of multiple group comparisons, given the number of derived trajectories, e.g. 5 trajectories give 4 group comparison results in adjusted alpha level of 0.01 (0.05 divided by 4).

To account for the non-random nature of the sample, all data were weighted to the proportions of gender, age, ethnicity, education and country of living obtained from the Office for National Statistics [15]. Analyses were conducted using Stata 16 and MPlus Version 8.4 [16].

#### 2.3.3 Secondary analyses

In the week of 23rd May, participants were asked to report their weight status prior to the lockdown. As these data were only available for a small proportion of participants (n = 6800), who also had data on eating behaviors, we decided to omit this variable from the main analyses to retain the largest possible sample size. Instead, we present the distribution of weight status across the different eating behavior trajectories as secondary exploratory analyses.

## 3 Results

### 3.1 Descriptive statistics

Descriptive statistics of the included sample at week commencing 28th March is summarized in Table 1, showing raw and weighted values.

**Table 1:** Raw and weighted descriptive statistics of the sample at week commencing 28th March (N = 22,374).

Week commencing 28th March	Raw	Weighted
	Percentage/Mean (SD)	Percentage/Mean (SD)
**Gender**		
Women	76%	51%
**Age**		
18–29 years	8%	20%
30–45 years	31%	26%
46–59 years	32%	24%
60 years	30%	30%
**Income**		
Low Income <30k, ref high income	43%	53%
**Education**		
GCSE or below	13%	33%
A-levels or equivalent	17%	34%
Degree or above	70%	34%
**Ethnicity**		
Ethnic minority, ref white ethnicity	5%	13%
**Marital status**		
Living with partner	65%	58%
Living without partner	6%	8%
Single	16%	22%
Divorced or widowed	13%	12%
**Mental health**		
Diagnosed mental illness	18%	19%
1 or more adverse life events	35%	39%
Low perceived social support (sum score≤18)	23%	28%
Loneliness score	4.81 (1.86)	4.98 (3.34)
General anxiety (GAD-7)	5.47 (5.22)	5.61 (9.56)
Depressive symptoms (PHQ-9)	6.42 (5.53)	6.99 (10.89)
**COVID-19 related worries**		
Minor/major worry about:		
Not getting enough food	39%	37%
Catching COVID-19	44%	44%
Getting seriously ill with COVID-19	48%	44%
**How would you describe your weight usually (i.e. before lockdown started)?** (N = 6800)		
Underweight	2%	2%
Normal weight	41%	39%
Slightly overweight	43%	45%
Very overweight	14%	14%

Notes: Abbreviations: GAD-7 = Generalized Anxiety Disorder assessment, PHQ-9 = Patient Health Questionnaire.

Responses to the eating behavior change questions during the first ten weeks of lockdown are presented in Supplement Fig. 1. Changes within participants are illustrated in Supplementary Fig. 2.

### 3.2 Description of trajectories

The model fit indices for different models including increasing numbers of trajectories are presented in Table 2. The five classes solution was deemed to be of best fit, with lowest AIC, BIC and adjusted BIC, and similar entropy to the four, six and seven classes solution, but avoiding trajectories smaller than 3% derived from solutions with more than 5 classes.

**Table 2:** Model fit indices for different model specifications, n = 22,374.

	−2 Log likelihood	AIC	BIC	adj BIC	entropy	N (%) of smallest trajectory
1 Class	175,945	206,934	206,966	206,953	NA	NA
2 Classes	−87945	175,907	175,971	175,945	0.84	5841 (26)
3 Classes	−82602	165,228	165,324	165,286	0.85	1989 (9)
4 Classes	−81247	162,527	162,655	162,604	0.81	1581 (7)
5 Classes	**−80652**	**161,345**	**161,505**	**161,442**	**0.79**	**1038** (**5**)
6 Classes	−80129	160,306	160,498	160,422	0.79	638 (<3)
7 Classes	−79718	159,492	159,716	159,627	0.79	274 (1)

Abbreviations: AIC = Akaike's Information Criterion, BIC= Bayesian Information Criterion, adj BIC = sample size adjusted Bayesian Information Criterion.

**Fig. 1:**
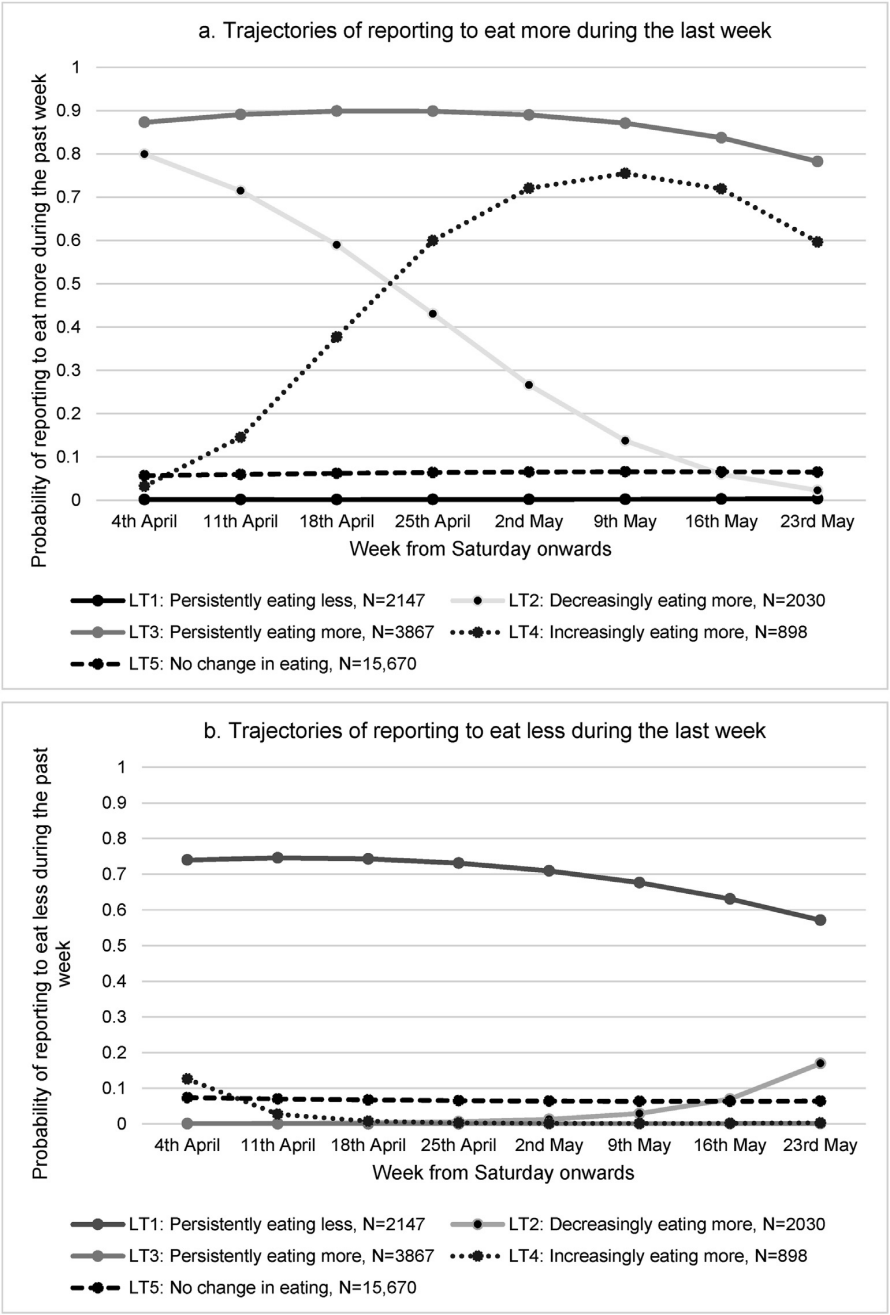
a & b. Estimated trajectories of change in from week starting Saturday 4th April to week starting Saturday 23rd May of stay at home lockdown measures, based on the five-class solution (N = 22,374).

These five trajectories are presented in Fig. 1a and b, showing the longitudinal change in “eating more in the past week” (Fig. 1a) and “eating less in the past week” (Fig. 1b), respectively. Overall, most of the participants, 64%, did not experience any change in eating throughout the observed period (LT 5: “No change in eating”). However, a smaller group of participants, 9%, reported persistently eating less (LT1: “Persistently eating less”). In contrast, 16% reported persistently eating more over the 8-week study period (LT3: “Persistently eating more”). The remaining two trajectories were marked by longitudinal change. Latent trajectory 2 consisted of participants, who reported eating more during the first weeks, but progressively decreasing the amount until week 8 (LT2: “Initial increase followed by steady decrease”, 8%). The smallest group of participants, 4%, did not report any changes in eating during the first week, but the food consumption increased substantially across time (LT4: “Increasingly eating more”).

### 3.3 Predictors of consistently eating less (LT 1)

The largest predictor of consistently eating less was experiencing one or more adverse life events during lockdown (OR = 1.69, SE: 0.16). Additionally, women (OR = 1.31, SE:0.13), as well as, people who were single (OR = 1.61, SE: 0.02), or divorced/widowed were also more likely to report eating less consistently throughout the observed lockdown period (OR = 1.72, SE: 0.23). Further, increased depressive symptoms were positively associated with this trajectory (OR = 1.11, SE: 0.02). However, having a university degree was found to be associated with lower odds of consistently eating less (OR = 0.64, SE: 0.10).

### 3.4 Predictors of decreasingly eating more (LT 2)

Women were more likely than men to eat more at the start of lockdown but for their eating behaviors to gradually return to normal as lockdown continued (OR = 1.82, SE: 0.17). Further, in comparison to participants older than 60 years, all younger participants were more likely to be in this group (aged 18–29: OR = 2.27, SE: 0.42; aged 30–45: OR = 2.02, SE: 0.25, and aged 46–59: OR = 1.68, SE: 0.19). Further, depressive symptoms were associated with higher odds of belonging to this group (OR = 1.10, SE: 0.01).

### 3.5 Predictors of consistently eating more (LT 3)

Women were also more likely to consistently eat more across lockdown (OR = 1.71, SE: 0.13), as were adults aged 30–45 years (OR = 1.57, SE: 0.16) in comparison to adults 60 years and older. Depressive symptoms were associated with higher odds of belonging to this group (OR = 1.14, SE: 0.01), as well as loneliness (OR = 1.07, SE: 0.02). Further, worries about getting ill were associated with greater odds of being in this group (OR = 1.36, SE:0.11).

However, participants with lower income (OR = 0.79, SE: 0.06) and a university degree were less likely to follow this trajectory (OR = 0.77, SE: 0.09). In addition, higher anxiety symptoms were associated with lower odds of consistently eating more during lockdown (OR = 0.97, SE: 0.01).

### 3.6 Predictors of increasingly eating more (LT 4)

Women were also more likely to progressively eat more during lockdown (OR = 1.74, SE: 0.24). Symptoms of depression were associated with a slightly higher odds of being in this class (OR = 1.09, SE: 0.02). However, participants with lower income (OR = 0.73, SE: 0.06) were less likely to follow this trajectory.

All results are listed in Table 3.

**Table 3:** Estimated odds ratios, standard errors, p-values for predictors of latent growth trajectories, in reference to the normative trajectory (LT5: no change in eating), N = 22,374.

	LT1: Persistently eating less vs reference			LT2: Decreasingly eating more vs reference			LT3: Persistently eating more vs reference			LT4: Increasingly eating more vs reference		
Predictors week starting 28th March	**OR**	**SE**	***p***	**OR**	**SE**	***p***	**OR**	**SE**	***p***	**OR**	**SE**	***p***
Women (ref men)	1.31	0.13	0.01	**1.82**	**0.17**	**<0.01**	**1.71**	**0.13**	**<0.01**	**1.74**	**0.24**	**<0.01**
18–29 yrs vs 60+ yrs	1.84	0.37	0.02	**2.27**	**0.42**	**<0.01**	1.33	0.20	0.09	0.62	0.18	0.03
30–45 yrs vs 60+ yrs	1.17	0.15	0.26	**2.02**	**0.25**	**<0.01**	**1.57**	**0.16**	**<0.01**	1.29	0.22	0.19
46–59 yrs vs 60+ yrs	1.20	0.14	0.15	**1.68**	**0.19**	**<0.01**	1.24	0.11	0.03	1.11	0.17	0.54
Low Income <30k, vs higher income	0.93	0.11	0.51	0.78	0.08	0.01	**0.79**	**0.06**	**<0.01**	**0.73**	**0.09**	**<0.01**
A-levels or equivalent vs GCSE or below	0.87	0.13	0.30	0.86	0.10	0.18	0.95	0.09	0.58	0.90	0.15	0.49
Degree or above vs GCSE or below	**0.64**	**0.10**	**<0.01**	0.80	0.09	0.02	**0.77**	**0.07**	**<0.01**	0.88	0.14	0.39
Ethnic minority (ref white)	1.45	0.28	0.10	0.96	0.17	0.83	0.98	0.16	0.91	1.26	0.35	0.45
Diagnosed mental illness	1.13	0.13	0.30	0.75	0.09	0.01	0.97	0.09	0.74	0.95	0.13	0.70
Diagnosed long-term health condition	1.12	0.10	0.24	0.93	0.08	0.35	0.91	0.06	0.18	1.19	0.15	0.21
Living without partner vs living with partner	1.59	0.25	0.02	1.00	0.19	0.98	1.05	0.15	0.73	1.12	0.25	0.64
Single vs living with partner	**1.61**	**0.20**	**<0.01**	0.78	0.10	0.03	0.81	0.09	0.03	1.15	0.18	0.41
Divorced or widowed vs living with partner	**1.72**	**0.23**	**<0.01**	1.10	0.14	0.47	1.05	0.11	0.66	1.68	0.35	0.05
1 or more adverse life events vs no adverse life events	**1.69**	**0.16**	**<0.01**	1.04	0.09	0.63	1.08	0.08	0.32	1.29	0.15	0.06
Loneliness score	1.05	0.03	0.08	1.02	0.03	0.45	**1.07**	**0.02**	**<0.01**	1.02	0.04	0.66
Low perceived social support (sum score ≤18, referent high)	1.28	0.16	0.07	1.12	0.12	0.32	1.11	0.10	0.25	1.25	0.19	0.18
General anxiety (GAD-7)	0.99	0.01	0.64	0.98	0.01	0.09	**0.97**	**0.01**	**<0.01**	1.03	0.02	0.19
Depressive symptoms (PHQ-9)	**1.11**	**0.02**	**<0.01**	**1.10**	**0.01**	**<0.01**	**1.13**	**0.01**	**<0.01**	**1.09**	**0.02**	**<0.01**
Worried about getting food	1.10	0.10	0.31	1.02	0.08	0.79	1.03	0.08	0.67	1.01	0.12	0.96
Worried about getting COVID-19	0.95	0.09	0.56	0.99	0.09	0.87	0.88	0.07	0.11	0.87	0.11	0.24
Worried about getting ill	1.18	0.12	0.14	1.03	0.10	0.75	**1.36**	**0.11**	**<0.01**	1.11	0.13	0.42

Abbreviations: OR = odds radio; SE= Standard error; BAME= Black Asian Minority English; GAD-7 = Generalized Anxiety Disorder assessment, PHQ-9 = Patient Health Questionnaire.

### 3.7 Secondary analyses

Self-reported weight status by eating behavior trajectory is reported in Table 4. These exploratory analyses suggest that there were more participants self-rated as very overweight in the consistently eating more trajectory (LT4) than in the group of participants experiencing no change in eating behavior (LT5) (29% versus 9%). In addition, participants reported to have underweight prior to lockdown were more common in the consistently eating less trajectory (LT1) versus any of the other groups (6% versus 0–2%).

**Table 4:** Self-reported weight status prior to lockdown by latent eating behavior trajectories, N (%).

Eating behavior latent trajectory	How would you describe your weight usually (i.e. before lockdown started)? asked from 23rd May, N = 6800			
	Underweight	Normal weight	Slightly overweight	Very overweight
**LT1**: Persistently eating less (N = 436)	24 (6%)	158 (41%)	167 (64%)	87 (17%)
**LT2**: Decreasingly eating more (N = 736)	710 (2%)	260 (36%)	329 (44%)	137 (18%)
**LT3**: Persistently eating more (N = 898)	12 (2%)	256 (24%)	429 (43%)	201 (27%)
**LT4**: Increasingly eating more (N = 194)	0 (0%)	116 (30%)	169 (47%)	78 (22%)
**LT5**: No change in eating behavior (N = 2362)	88 (2%)	1983 (42%)	1815 (46%)	481 (10%)

## 4 Discussion

Around 36% of this UK cohort experienced changes to their eating behaviours during the COVID-19 pandemic. This estimate is lower than that reported from another UK-based online survey (75%; n = 559) of overall food intake in response to lockdown measures [17]. However, our study provided novel insight not just into a cross-sectional assessment of change but an understanding of the trajectories of that change. Specifically, we were able to generate five distinct groups of eating behavior trajectories over 8 weeks: *“Persistently eating less*”, “*Initial increase followed by steady decrease*”, *“Persistently eating more”*, “*Increasingly eating more*”, and “*No change in eating”*. Drastic changes in amounts eaten are detrimental to health, irrespective of whether habitual dietary intake is optimal for weight maintenance or not [18]. Therefore the findings that many individuals did experience changes to their food intake is of concern as such changes can also impact overall dietary quality and, further down the line, bodyweight management [19].

In considering who was at greatest risk of this, we identified several subgroups. Younger adults were more likely to report changes in eating behaviours, echoing results from a pre- and post-lockdown meta-analysis of cross-sectional findings from five national UK-based cohorts [20]. Middle-aged participants (30–59 years) were likely to eat more during lockdown, which might represent their potential greater financial stability and job security: food may have acted more as a source of comfort and part of household routine [21]. Our results also suggested that women were more likely than men to report significant changes, as was reported by a number of cross-sectional studies [17,22,23].

A pre-existing mental or physical health condition was not a predictor of eating behavior change. However, current depressive symptoms were associated with an increased risk of experiencing any changes in eating behavior, which corresponds with cross-sectional data during COVID-19 [17,22,23]. Changes in appetite are core symptoms of depression [24], and research showing that having overweight is associated with depression [25]. We also found associations between persistently eating more and loneliness, which reflects another commonly observed association between loneliness and obesity [26]. One potential behavioral link is emotional eating: the tendency to self soothe and reward oneself with palatable foods during times of negative emotions. However, our study could not elucidate if depression was the driver of emotional eating or a co-symptom of the psychological challenges being experienced.

Higher educational attainment was found to be a protective factor against persistent change in eating behaviors. This is possibly explained by greater recognition of changes in dietary patterns, greater self-regulatory capacity, or better mitigation of drastic changes to maintain healthier eating intentions [27,28]. On the other hand, low income was a protective factor against eating more as lockdown continued, likely the result of the financial challenges experienced. There have been several reports that the economic fallout of the COVID-19 pandemic has more than quadrupled food insecurity prevalence to 16% in the UK [29]. Notably, being worried about getting food was however not associated with changes in eating behaviors in the present study. This might be an indication that some people were able to turn to food banks to meet their usual dietary energy needs through these challenging times, and that food availability in supermarkets, online and in-store, rapidly stabilized. These data, however, do not reflect other changes to diet, which may have occurred because of financial hardship during lockdown, such as an overreliance on unbalanced meals or less nutrient dense foods.

Interestingly, one group (9% of the sample) reported consistently eating less over the studied period. The fact that some people respond to a stressful situation by eating less has previously been observed, and there are large individual differences in the way which people's appetite change when exposed to stress [30,31]. Participants living by themselves, as well as participants reporting more than one stressful life event (e.g bereavement) were more likely to fall into this group. Notably, lockdown measures have been linked to disordered eating behaviors commonly seen in eating disorders, such increased restriction of food intake and binge eating behaviors [32]. Further, patients with eating disorders have described lockdown as detrimental, reporting disruption of routines and decreased feelings of control which increased worries about their illness [33].

Another unique finding was the identification of two dynamic eating behavior trajectories (“Initial increase followed by steady decrease”, and “Increasingly eating more”). This could indicate that some individuals gradually learned to buffer the stress and uncertainty of lockdown in other ways than changing their eating behaviors. Indeed, eating more sweet foods during lockdown has been identified as a psychological coping strategy [19]. Alternatively, individuals may have increasingly struggled to self-regulate their eating behaviors as the length of lockdown and its related pressures increased.

In our exploratory analyses we found associations between changes in eating behaviors and pre-lockdown weight. Participants self-reporting as being "very or slightly overweight" prior to lockdown formed the majority of the participants (70%) following the “consistently eating more” trajectory. This echos findings from other studies showing that people with a higher BMI experienced more barriers to weight management, and could be due to lower motivation and perceived control over food intake [34] and lower adherence to healthy eating patterns [3].

### 4.1 Strength and limitations

This study has several strengths including its large sample size, the longitudinal tracking of participants, and the inclusion of measures on psychological and social experiences during the COVID-19 pandemic. However, there are several limitations to consider. The study is not based on a nationally representative sample, although it does have good stratification across all major socio-demographic groups and analyses were weighted based on population estimates of key demographic characteristics. It is therfore possible that more extreme experiences were not adequately captured. Change in eating behaviors was ascertained using a one-item measure so analyses are limited in detecting nuanced changes in eating behaviors. The phrasing of the question was also posed in a relative, subjective manner comparing change to the individuals ‘usual’ amount eaten. It is therefore not possible to objectively know if individuals ate more or less than recommended daily food intake, or if changes was self-perceived. But more onerous measures like 24-h diet recalls were not viable in the current study. In addition, the measure did not capture what or why participants were eating, and thus it was not possible to assess if overall dietary quality was affected by lockdown. Even though we included a large range of predictor variables, we failed to detect predictors for all the trajectories (especially LT2 and LT4), which implies that potentially important factors were not captured. Further, data collection did not include measure of weight status from the beginning, and the question about weight status prior to lockdown was only included from the 23rd May onwards. Therefore, our secondary analyses included only a smaller subsample. In addition, this weight related question was highly subjective and did not involve objective measures such as height and weight. Self-report measure of weight status may be unreliable [35], so future research would benefit from objective weight measures.

## 5 Conclusions

Whilst the magnitude of the psychological and physiological aftermath for health systems remains uncertain, it is nevertheless concerning that many groups who experienced changes to their eating behaviours are those who were already more vulnerable, which suggests there may have been an exacerbation of inequalities in health behaviors and health outcomes during COVID-19 [36]. Further support is therefore needed as the pandemic continues and in its aftermath to protect people from the risk of obesity or eating disorders [7,37]. Public health interventions face the challenge of informing at risk individuals how to stay healthy, without exacerbating stigma and weight-related anxiety [38], while protecting people with eating disorders [39]. Nevertheless, interventions such as online support groups to increase social connection amongst people experiencing changing eating habits could prove valuable [40], as could the provision of more remote consultations to continue care, and the use of smartphone applications relating to nutrition, weight and diabetes, to mitigate the detrimental effects of stress and anxiety from negatively affecting nutritional health and body weight. A streamlined response to COVID-19 in the context of nutrition, obesity and eating disorders is important to optimize public health outcomes and reduce the impacts of this pandemic on individuals, vulnerable groups, and society, but also to prepare for potential future epidemics.

## Ethics approval and consent to participate

Ethical approval for the COVID-19 Social Study was granted by the UCL Ethics Committee. All participants provided fully informed consent. The study is GDPR compliant.

## Availability of data and materials

Data is from the Covid Social Study which is currently not open, but interested collaborators may contact the study team, www.covidsocialstudy.org.

## Competing interests

All authors declare no conflicts of interest.

## Funding

This Covid-19 Social Study was funded by the 10.13039/501100000279Nuffield Foundation [WEL/FR-000022583], but the views expressed are those of the authors and not necessarily the Foundation. The study was also supported by the MARCH Mental Health Network funded by the Cross-Disciplinary Mental Health Network Plus initiative supported by 10.13039/100014013UK Research and Innovation [ES/S002588/1], and by the 10.13039/100004440Wellcome Trust [221400/Z/20/Z]. DF was funded by the 10.13039/100010269Wellcome Trust [205407/Z/16/Z]. The researchers are grateful for the support of a number of organisations with their recruitment efforts including: the UKRI Mental Health Networks, Find Out Now, UCL BioResource, SEO Works, FieldworkHub, and Optimal Workshop. The study was also supported by, HealthWise Wales the Health and Care Research Wales initiative, which is led by Cardiff University in collaboration with SAIL, Swansea University. The funders had no final role in the study design; in the collection, analysis and interpretation of data; in the writing of the report; or in the decision to submit the paper for publication. All researchers listed as authors are independent from the funders and all final decisions about the research were taken by the investigators and were unrestricted. MH is funded by fellowship from the 10.13039/501100000265Medical Research Council UK [MR/T027843/1].

## Authors' contributions

The author responsibilities wereas follows: MH, ADS, FB: Study design; AS, DF: Data acquisition; MH, ADS, FB, AS, DF: Analyses and interpretation; MH, ADS: Drafting of the manuscript; MH, ADS, FB, AS, DF: Critical revision of the manuscript, and all authors read and approved the final manuscript.

## Data share statement

Data described in the manuscript will not be made available as data from the Covid Social Study which are currently not publicly available, but interested collaborators may contact the study team. Analytic code will be made available upon request and will be freely available and without restriction on the Covid Social Study github page.
