# Supplementary material for: Trajectories of eating behavior during COVID-19 lockdown: Longitudinal analyses of 22,374 adults
Source: Clin Nutr ESPEN. 2021 Apr;42:158–65. doi: 10.1016/j.clnesp.2021.01.046 (PMC7871880; doi:10.1016/j.clnesp.2021.01.046)
Supplement: Multimedia component 1 [file mmc1.docx]

**Online Supplementary Material**

Supplementary Table 1: Comparison of items in the original and revised Perceived Social Support Questionnaire (F-SozU K-6).

| **Original** | **Adapted for COVID-19**  In the past week, I feel… |
| --- | --- |
| I experience a lot of understanding and security from others | I have experienced a lot of understanding and support from others |
| I know a very close person whose help I can always count on | I have a very close person whose help I can always count on |
| If necessary, I can easily borrow something I might need from neighbours or friends | If necessary, I can easily borrow something I need from neighbours or friends |
| I know several people with whom I like to do things | I have people with whom I can spend time and do things together |
| When I am sick, I can without hesitation ask friends and family to take care of  important matters for me | If I get sick, I have friends and family who will take care of me |
| If I am down, I know to whom I can go without hesitation | If I am feeling down, I have people I can talk to without hesitation |

Supplement Figure 1. Responses on eating behavior question across 8 weeks from between week starting 4^th^ April and week starting 23^rd^ May 2020.

Supplement Figure 2. Lasagne Plot of responses to eating behavior question between week starting 4^th^ April and week starting 23^rd^ May 2020
